# Some Studies in Occlusion

**Published:** 1905-03

**Authors:** Edward H. Angle

**Affiliations:** St. Louis, Mo.


### SOME STUDIES IN OCCLUSION.[1]

BY EDWARD H. ANGLE, M.D., D.D.S., ST. LOUIS, MO.

MR. PRESIDENT, MEMBERS OF THE ACADEMY OF STOMATOLOGY, AND FRIENDS,—The subject of my paper this evening, as announced, is "Orthodontia," but I am going to take the liberty of modifying this title to one which I think will awaken a keener interest and give a broader understanding of the term "Orthodontia." I shall call it "Some Studies in Occlusion," for I feel that a greater interest should be awakened among you all to that principle which goes back of orthodontia and underlies its very inception and entirety, and not only orthodontia, but every operation which you as dentists are ever called upon to perform upon the teeth. This principle is occlusion, and I will define occlusion as being the normal relations of the inclined occlusal planes of the teeth when the jaws are closed.

I hope that at some day not far distant I may be able to read in the announcement of our colleges that a new chair has been added, a new professorship established, the mightiest of them all,—the chair of the Science of Occlusion,—where the student from the time he enters the school shall be taught occlusion broadly, deeply, grandly, and not only the one link in the chain of occlusion,—the human teeth,—but comparative occlusion from the lowest to the highest. Then he will be impressed with its mighty importance, and so studied, this knowledge of occlusion will be a mighty incentive in governing the plan and performance of his every operation upon the teeth. For were the teeth not created solely for occlusion? Study their shapes, study their forms and proportions, examine them microscopically, study their positions and periods of eruption, and the very structure and arrangement of that wonderful membrane that holds them in position, and, too, that peculiar structure, the alveolar process, that comes at their bidding and vanishes with their going,—all, *all* point to occlusion and the one grand object of its function. Who can estimate the value of normal occlusion in the development and maintenance of the entire physical

---

[1] Read before the Academy of Stomatology, Philadelphia, March 1, 1904.

economy, even reacting on the developing masticatory apparatus itself; or who can estimate the results on the development of the entire physical economy when handicapped by malocclusion? I know you will lightly pass this thought by, but I assure you it is not one to be lightly cast aside by truly scientific men, inasmuch as malocclusion is rapidly growing to be the rule instead of the exception.

When men shall have become impressed with the importance and value of occlusion, then will many questions in dentistry which now agitate and provoke controversy be naturally and scientifically settled. Then will the practice of placing bridges on leaning piers, which in reality serve chiefly the purpose of spanning space and for the lodgement of débris, be changed, and bridges will be constructed for purposes of occlusion and attached to piers that have been first placed from a leaning position to that best suited for occlusion and support. When men shall understand the real meaning of occlusion then will there be vastly fewer bridges and crowns needed, for the jewels in the crown of occlusion will be prized more nearly in accordance with their true worth, and the earliest and most careful attention will then be given, not only to their preservation, but to their eruption and direction into normal positions. Then will their careless sacrifice surely be regarded as a punishable crime, for I believe that, generally speaking, the loss of a first lower molar is really a far greater damage to the physical economy, as a whole, than would be the loss of a finger.

When men shall understand occlusion they will realize that the loss of a tooth is of such serious consequence in its relation to the rest of the dental apparatus that it should and must be immediately substituted by the best that is artificial.

Then the question of extraction for the correction or prevention of malocclusion will be naturally and thoroughly settled of itself, and we shall read with amazement and view with surprise the pictures and discussion of cases treated by orthodontists of these days when extraction was resorted to and defended with all complacency.

When men shall know occlusion, then will the requirement of the natural form to each filling be self-evident, and such monstrosities of form in fillings and crowns as those we now daily see coming fresh from the hands of reputable operators will be regarded with amazement.

When men shall be taught and shall have mastered occlusion then will orthodontia no longer be made subservient to all else in colleges and practice, but it will take its rightful position as the first, the principal, and the most important effort toward the betterment of the permanent human teeth, for then all will be impressed with the mighty importance of teeth being directed into their normal relations, and that, too, not at the age of sixteen, but at the very time of their eruption, for then will it become self-evident that that time is the golden time for orthodontia and the highest possible betterment of the relations of the teeth, and how strange will such advice sound, now so commonly given by dentists to anxious mothers, as "Your child is too young," or, "Wait until all of the teeth are in position." When men shall have mastered occlusion they will realize that perfect fillings can only be made when teeth are in perfect relations, and that the perfect contouring of a filling is essential, not only to the immediate locality of each tooth, but, likewise, to the bracing and maintaining of all the other inclined planes in both dental arches, and that to sacrifice a cusp or any considerable portion of a tooth in a denture normally occluded is soon to result in the modifying of the beautiful, the harmonious, the efficient relations of many if not all of the remaining inclined planes. Then will such questions as "extension for prevention" have a true light thrown upon them by many not now comprehended.

When our new professorship is established,—I mean a *real* professorship with a *real* professor,—then will anatomy and histology have a different meaning to the students. The study of the development of the jaws and the alveolar process will then have a real meaning, as well as that of the pathology of the nose and throat, for their mighty bearing upon occlusion will then be comprehended and its teaching be listened to by students in a different spirit. Then the relation of the dentist and the true rhinologist will not be that of casual acquaintance, but of hearty friendship and co-operation.

Then will the pale-faced, badly nourished mouth-breather, with perhaps only his second molars occluding, now more commonly seen than children with strabismus were thirty years ago, be as rare as these children now are, for since the eye has been scientifically studied and treated at the proper age this formerly common ailment has been largely overcome.

I am also convinced that the study of occlusion goes beyond the study of dentistry, and is a wonderful impetus to the appreciation and understanding of art, for no one can study those wonderful curves and lines of beauty and harmony of proportion of the teeth in normal occlusion, and what this means to the general balance of the rest of the face, without having a keener appreciation of art, for what is art but harmony of proportion of form and color? and I sometimes think that the reason that dentists seem to have such poor ideas of art is their lack of an ideal to work to. Or, in other words, from the nature of things, as dentistry is now practised, the correct form of a tooth and its importance means but little to the dentist. The picture of the surface to be restored is imperfect in his mind, and the malposition of the tooth renders the perfect form of his filling ofttimes impossible, so he works upon an imperfect surface and aims to stop decay, and leaves a surface without regularity of form.

I know that I shall be accused of being an idealist. I hope so, for there is need of a few more in dentistry. I shall be accused of advocating the practice of that which is too ideal and impossible. You will say that we have to work for people who have passed the age for consideration of the ideal or normal in occlusion, and that which will prolong the usefulness of the organs of mastication is all that can be considered. I grant you that this is probably true with many of your patients, but is it not also true that almost daily those youthful patients come to you in whom the golden hours for establishing the normal in occlusion is passing, and are you treating them according to the demands of occlusion? Or are you treating them without consideration for the matchless form of grace and beauty of the surfaces you are restoring, and of what those surfaces mean in that dental apparatus and to that face as a whole? Are you seeing to it that the inclined occlusal planes lock and harmonize in the manner designed by nature for the best use, preservation, and beauty of an harmonious whole? If not, you are neglecting your highest and most sacred duty as dentists, for the period for the normal locking of the cusps of these teeth is one that only comes once, and if you have neglected your duty in the intelligent supervision of this locking and have permitted the beginning of chaos in occlusion you surely are not true to your trust.

In this paper I shall have little to say regarding regulating appliances, for in comparison with occlusion they seem so lacking in importance that I feel it were probably better not to intrude them upon your time, for only after occlusion is comprehended and thoroughly mastered can the proper form and use of a regulating appliance have any true meaning. When occlusion shall be mastered then will the question of concentration or specialization naturally be settled, for then even the colleges will learn that it were far wiser and better to so educate students that they might in their life's work do some one thing well than many badly, as is now the rule. Then will the different branches of dentistry be truly specialized, and occlusion will be the basis of them all and the bond that will bind them all into one harmonious whole.

In the pictures which I have prepared for this evening many are new. Some have been seen by a few of you before, but all have been made to try to tell in a rambling way the story of occlusion, a story which ought to be very old,—which is old in truth,—but one that is far too new to many of those who are intrusted with the care of the teeth.

Following this Dr. Angle showed one hundred and thirty slides of the occlusion and faces of patients treated for malocclusion of their teeth.

